# Fecal bacterial microbiome diversity in chronic HIV-infected patients in China

**DOI:** 10.1038/emi.2016.25

**Published:** 2016-04-06

**Authors:** Yang Sun, Yingfei Ma, Ping Lin, Yi-Wei Tang, Liying Yang, Yinzhong Shen, Renfan Zhang, Li Liu, Jun Cheng, Jiashen Shao, Tangkai Qi, Yan Tang, Rentian Cai, Liqian Guan, Bin Luo, Meiyan Sun, Ben Li, Zhiheng Pei, Hongzhou Lu

**Affiliations:** 1 Department of Infectious Diseases, Shanghai Public Health Clinical Center, Fudan University, Shanghai 201508, China; 2 Department of Laboratory Medicine, Shanghai Guanhua Hospital, Shanghai 200065, China; 3 Shanghai TargetDrug Ltd., Shanghai 201202, China; 4 Center for Synthetic Biology Engineering Research, Shenzhen Institutes of Advanced Technology, Chinese Academy of Sciences, Shenzhen 518055, China; 5 Clinical Laboratory, Shanghai Mental Health Center, Shanghai Jiao Tong University School of Medicine, Shanghai 200065, China; 6 Clinical Microbiology Service, Department of Laboratory Medicine, Memorial Sloan-Kettering Cancer Center, New York, NY 10065, USA; 7 Departments of Pathology and Medicine, New York University School of Medicine, New York, NY 10016, USA; 8 The Department of Veterans Affairs New York Harbor Healthcare System, New York, NY 10010, USA

**Keywords:** antiretroviral treatment, feces, HIV, microbiome, 16S rRNA

## Abstract

The purpose of this study was to identify fecal bacterial microbiome changes in patients with chronic human immunodeficiency virus (HIV) infection in China. Bacterial 16S rRNA genes were amplified, sequenced (454 pyrosequencing), and clustered into operational taxonomic units using the QIIME software. Relative abundance at the phylum and genus levels were calculated. Alpha diversity was determined by Chao 1 and observed-species indices, and beta diversity was determined by double principal component analysis using the estimated phylogeny-based unweighted Unifrac distance matrices. Fecal samples of the patients with chronic HIV-infection tended to be enriched with bacteria of the phyla *Firmicutes* (47.20%±0.43 relative abundance) and *Proteobacteria* (37.21%±0.36) compared with those of the non-HIV infected controls (17.95%±0.06 and 3.81%±0.02, respectively). Members of the genus *Bilophila* were exclusively detected in samples of the non-HIV infected controls. *Bacteroides* and *arabacteroides* were more abundant in the chronic HIV-infected patients. Our study indicated that chronic HIV-infected patients in China have a fecal bacterial microbiome composition that is largely different from that found in non-HIV infected controls, and further study is needed to evaluate whether microbiome changes play a role in disease complications in the distal gut, including opportunistic infections.

## INTRODUCTION

The human gut is home to a complex microbial ecosystem or microbiome that is composed of thousands of species. The total bacterial population in the gut exceeds 1 × 10^14^ cells, which is approximately ten times greater than the number of human cells.^1^ This large and diverse community of bacteria is known to play key roles in modulating host metabolism and immunity.^2^ To better understand the role of these microbes in the human body and to determine the potential effects of fecal bacterial microbiome composition on disease susceptibility, extensive microbial diversity surveys have been conducted.^3^ With the recent development of next-generation sequencing methods, these surveys have revealed a number of global patterns of bacterial diversity in human intestinal ecosystems.^4^

The composition of the fecal microbial microbiome is unique for each individual, depending, to some extent, on the environment and diet of the host. Although this ecosystem can evolve, a stable balance must be maintained.^5^ Using barcode pyrophosphate sequencing technology, Young *et al.*^6^ found that the intestinal microbiome is clearly different among the South Koreans, Americans, Chinese and Japanese; however, these individuals were all considered healthy, indicating that a stable or a balanced fecal bacterial microbiome is likely a greater indicator of intestinal health than the actual composition of the biome. Notably, this delicate balance of the microbiome can be disrupted in a number of situations, including illness.^7^ For example, in a recent high-throughput diversity and functional analysis, Zoetendal *et al.*^8^ observed that opportunistic infections may be associated with detrimental changes in the intestinal microbiome. Furthermore, through analysis of >400 species of microbes, Rajilić-Stojanović *et al.*^9^ found that the most abundant microbes present in the gut were also the most susceptible to change. In addition, various profiles have been shown to be present in the microbiome of elderly individuals with low immunity and people with chronic diseases, further highlighting a link between altered gut bacteria and health complications in the host. However, a thorough analysis of the fecal bacterial microbiome in patients with chronic human immunodeficiency virus (HIV) infection, particularly Chinese patients, has not been performed to date.

Acquired immune deficiency syndrome is a serious chronic infectious disease caused by HIV. Although the survival rate for Acquired immune deficiency syndrome/HIV patients has significantly improved since the introduction of antiretroviral therapy (ART),^10, 11^ persistent disease progression and clinical complications in virally suppressed individuals are suggestive of the contributions of factors other than HIV replication alone. Notably, a recent study indicated that antibacterial treatment can affect the normal fecal microbiome of chronic HIV-infected patients, which may provide an opportunity for pathogenic bacteria to multiply and trigger infection and other complications.^11^ Similar studies have primarily focused on patients in Europe and the United States,^12, 13, 14^ whereas the role of the fecal bacterial microbiome in disease progression in Chinese chronic HIV-infected patients is largely undocumented. In recent years, we have observed an increase in the number of patients living with HIV in the Shanghai Public Health Clinical Center. Many of these patients complain of abdominal discomfort, intermittent diarrhea and even persistent diarrhea after ART, which are complications that tend to occur repeatedly and do not appear to have a common cause. We suspect that these abdominal issues are related to changes in the intestinal microbiome of chronic HIV-infected patients. To test this hypothesis, we employed 454 pyrosequencing and the QIIME pipeline to explore microbial diversity in chronic HIV-infected patients and non-HIV infected controls and to determine the possible role of the gut ecosystem in the global health of the host during ART.

## MATERIALS AND METHODS

### Subjects and sample collection

In this study, we enrolled 13 patients with chronic HIV infection who had recently been admitted to the Shanghai Public Health Clinical Center. All patients except two received ART. Of the 13 participants, pulmonary infection was found in six; combined pulmonary infection, CMV infection and non-tuberculosis mycobacteria, in one; combined pulmonary infection and non-tuberculosis mycobacteria, in one; amebiasis combined with pyogenic liver abscess, in one; lymphoma, in one; fungal infection combined with pulmonary infection, in one; combined pulmonary infection and syphilis, in one; and pyogenic liver abscess, in one (Table 1). Of these, 69.2% were male (nine subjects) and 30.8% were female (four subjects). The median age was 47 years (range: 22–72 years). The diagnosis of chronic HIV-infection was made according to known diagnostic standards.

In addition, four non-HIV infected controls ranging from 29 to 71 years of age were enrolled in this study. Of these control subjects, three patients (DB3, DP3 and DP4) were suffering from diarrheal disease diagnosed at the outpatient services at Shanghai Tongji Hospital, whereas a single healthy control was referred from the Institute of Medicine Science, Shanghai Tongji Hospital. A basic outline of the patient characteristics can be found in Table 1.

One fecal sample was obtained from each subject. From the healthy subject, three additional samples were obtained (four total), one per week over the course of a month, for use as internal quality controls. All of the fecal samples were properly handled and collected in disposable plastic sterile dung cups. All samples arrived at the laboratory within 24 h and were immediately frozen at -20 °C and stored until analysis. This study was approved by the Ethics Committee of the Shanghai Public Health Clinical Center affiliated with Fudan University, China.

### DNA extraction

DNA isolation with the QIAamp DNA Stool Mini Kit (Qiagen, Duesseldorf, Germany) was performed in accordance with the manufacturer's instructions (QIAamp DNA Stool Handbook 04/2010, Germany). DNA concentrations were measured at 260 nm with a NanoDrop 2000 spectrophotometer (Thermo Scientific, Waltham, MA, USA).

### PCR enrichment of the V3–V4 region, pyrosequencing and statistical analysis

Details concerning the PCR methodology utilized in this study can be found in Chakravorty *et al.*^15^ Twenty pairs of barcoded primers were used for the enrichment of the bacterial 16S rRNA gene V3–V4 hypervariable regions.^15, 16, 17, 18^ The enriched V3–V4 regions were pooled, mixed and sequenced using the 454 FLX pyrosequencing platform (Roche 454 GS FLX, Basel, Switzerand).

Following sequencing, the reads were de-multiplexed into samples according to the barcodes using the QIIME software (Denver, CO, USA) pipeline,^19^ with the default parameters. Primer and barcode sequences were removed. Operational taxonomic unit clustering was performed at a 97% similarity threshold using the QIIME pipeline. The relative abundance of the taxa at the phylum and genus levels were calculated. The diversity was estimated with Chao 1 and observed-species indices calculations (alpha or within-sample diversity) and double principal component analysis (DPCoA; beta or between-sample diversity) using the phylogeny-based unweighted Unifrac distance matrices. The difference between specific taxa was analyzed using analysis of variance (ANOVA) followed by *post-hoc t*-tests, with Bonferroni and Benjamini-Hochberg false discovery rate (FDR) corrections for multiple testing. *P*<0.05 and *q*<0.1 were considered significant. Linear discriminant analysis effect size (LEfSe) analysis was used to identify different taxa between the cases and the controls.^20^

## RESULTS

**Figure 1:**
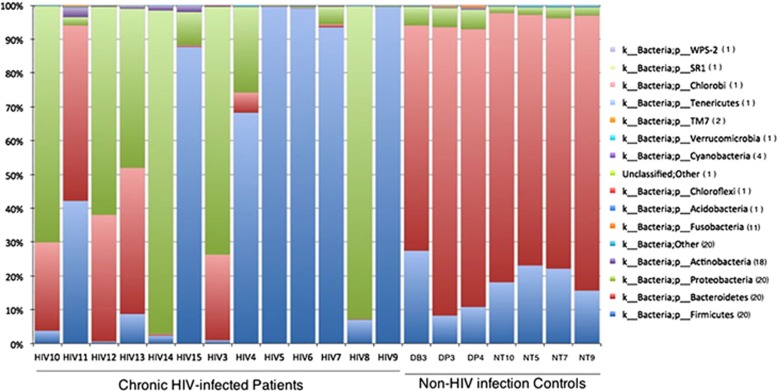
Phylum-level taxon distribution in chronic HIV-infected patients and non-HIV infected controls. Stacked columns for each of the 20 samples from the 17 subjects enrolled in this study show the abundance of a given phylum as a percentage of the total bacterial sequences in the sample. All 16 phyla (one unclassified) with an abundance of at least 0.1% in at least one subject were included. Numbers in parentheses in the legend indicate the number of samples containing the taxon.

**Figure 2:**
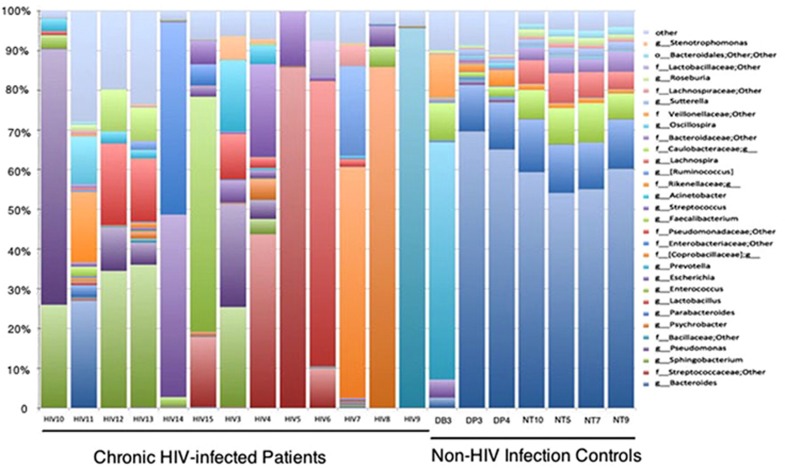
Genus-level taxon distribution in chronic HIV-infected patients and non-HIV infected controls. Stacked columns for each of the 20 samples from the 17 subjects enrolled in this study show the abundance of a given genus as a percentage of the total bacterial sequences in the sample. All 31 genera with an abundance of at least 0.1% in at least one subject were included.

The 20 fecal samples yielded 376 627 high-quality reads. On average, each sample yielded ~18 811 sequences, ranging from 1393 to 76 583. Overall, 16 unique bacterial phyla (Figure 1) or 273 genera were identified in the 20 samples, with an average of 61 genera in each sample (Figure 2).

### Diversity indices

**Figure 3:**
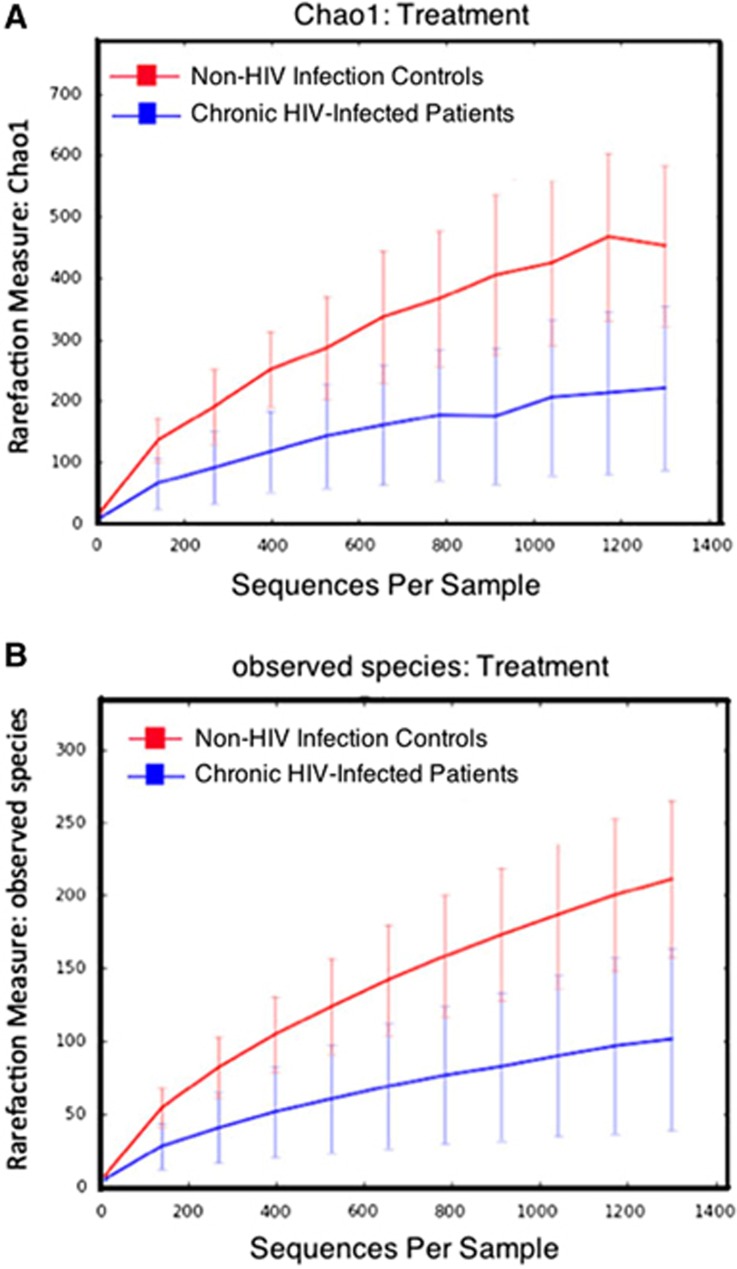
Analysis of alpha diversity in chronic HIV-infected patients. Diversity indices in chronic HIV-infected patients (blue) versus non-HIV infected controls (red) predicted diversity by Chao 1 (**A**) and observed diversity (**B**).

**Figure 4:**
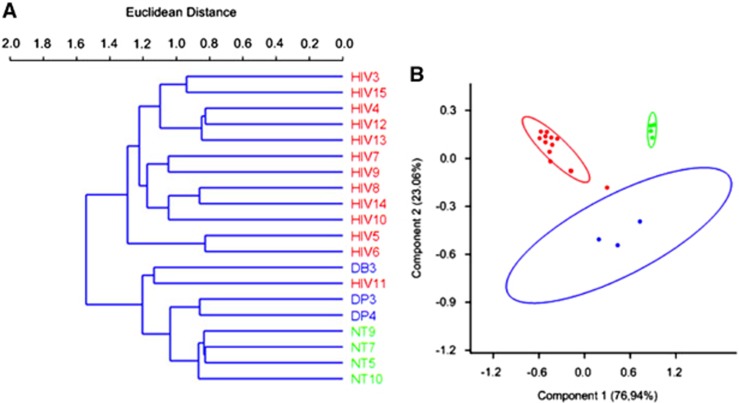
Analysis of beta diversity in chronic HIV-infected patients. Cluster dendrogram (**A**) illustrating the bacterial diversity of chronic HIV-infected patients (red) compared with non-HIV infected controls (blue and green). Double principal component analysis (**B**) of fecal microbiome composition in chronic HIV-infected patients (red) compared with non-HIV infected controls (blue for the non-HIV subjects with diarrhea and green for the healthy controls).

Alpha diversity was determined using Chao 1 and observed-species index calculations (Figure 3, panels A and B, respectively). These data indicated that HIV infection was associated with a decrease in microbiome diversity. Beta diversity was determined using DPCoA with the phylogeny-based unweighted Unifrac distance matrices and revealed a clear separation in bacterial community composition between the controls and the HIV-infected patients (Figure 4).

### Difference in specific taxa between chronic HIV-infected patients and non-HIV infected controls

**Figure 5:**
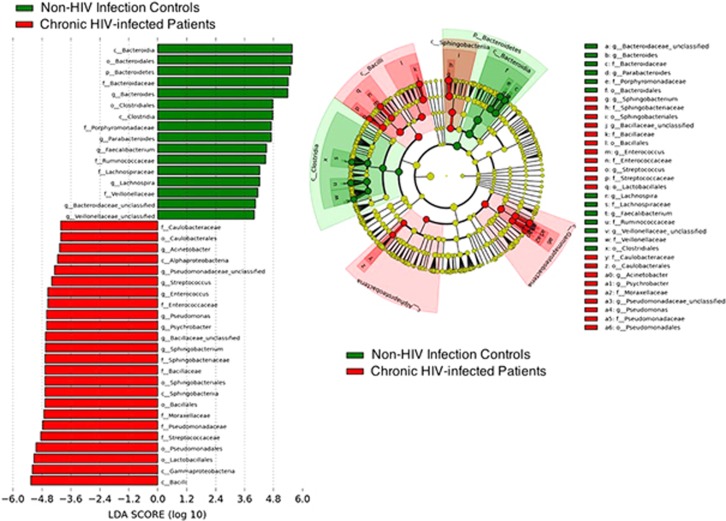
Differential representation of bacterial lineages by LEfSe between chronic HIV-infected patients and controls. Histograms of enriched bacterial taxa in HIV-infected patients are displayed in red, and in controls, in green.

To examine these differences more closely, we investigated microbial distributions at the phylum and genus levels. At the phylum level, the data indicated that the chronic HIV-infected patients tended to be enriched with *Firmicutes* (47.20 versus 17.94% *q*=0.16, *P*=0.10) and *Proteobacteria* (37.21 versus 3.81% *q*=0.06, *P*=0.03) and depleted in *Bacteroidetes* (17.48 versus 77.58% *q*=9.13E-07, *P*=1.52E-07) (Figure 1 and Supplementary Table S1) compared with the non-HIV infected controls. The distribution of bacteria according to the genus in each sample showed that several genera were different in abundance between the two groups (Figure 2). We further investigated intergroup differences by using ANOVA and *post-hoc* tests, and *post-hoc P*-values were FDR-corrected (*q-*values) (Table 2). The data indicated a high level of variability between the fecal microbiome compositions of the chronic HIV-infected patients and the non-HIV infected controls at the genus level (*P*=0.001*, R*=0.7813). The control subjects appeared to have significantly higher levels of bacteria from the genera *Bacteroides* (52.30% versus 2.16% *q*=3.17E-05, *P*<0.0001), *Parabacteroides* (10.50 versus 0.25% *q*=2.76E-05, *P*<0.0001), *Faecalibacterium* (6.54 versus 0.19% *q*=5.67E-05, *P*<0.0001), *Sutterella* (1.52 versus 0.02% *q*=1.96E-05, *P*<0.0001), *Phascolarctobacterium* (7.96 versus 9.81E-03% *q*=2.40E-05, *P*<0.0001), *Roseburia* (1.17% versus 1.36E-01% *q*=3.90E-03, *P*<0.001), *Lachnospira* (3.57% versus 5.73E-02% *q*=6.76E-03, *P*<0.05) and unclassified *Bacteroidaceae* (2.37 versus 6.84E-02% *q*=5.66E-03, *P*<0.05) compared with the chronic HIV-infected patients (Table 2, Figure 5). It is also worth noting that bacteria of the genus *Bilophila* appeared only in the non-HIV infected controls.

These findings are shown in the LEfSe analysis (Figure 5). Taken together, these results imply that an entirely different bacterial ecosystem is present in the feces of chronic HIV-infected patients compared with non-HIV infected controls with gastrointestinal illnesses and healthy individuals (Supplementary Table S2).

## DISCUSSION

To elucidate the potential role of the fecal microbiome in HIV disease progression, we compared the fecal bacterial microbiome of chronic HIV-infected patients with that of non-HIV infected controls. We found that chronic HIV-infected patients tended to be enriched with bacteria of the phyla *Firmicutes* and *Proteobacteria.* Our observations are consistent with those of a recent meta-analysis showing that the phylum *Proteobacteria* is highly abundant in the intestinal lumen of patients suffering from chronic HIV infection.^21^

The observed phylum level changes can be traced to lower ranked taxa. Within *Firmicutes,* a phylum dominated by Gram-positive bacteria, the changes in relative abundance were most prominent for the genera *Phascolarctobacterium, Faecalibacterium, Roseburia* and *Lachnospira,* as well as for taxa within the *Veillonellaceae*, *Ruminococcaceae* and *Lachnospiraceae* families in the order *Clostridiales,* class *Clostridia*. Within the *Proteobacteria,* a phylum of Gram-negative bacteria, the difference in relative abundance between the chronic HIV-infected patients and the non-HIV infected controls was most prominent for the genera *Bilophila* and *Sutterella*, which belong to the class *Betaproteobacteria,* as well as for others from the class *Deltaproteobacteria*, including taxa in the *Alcaligenaceae* and *Desulfovibrionaceae* families in the orders *Burkholderiales* and *Desulfovibrionales*, respectively.

In addition to the bacterial enrichment observed in the chronic HIV-infected patients, several bacterial taxa were depleted in the HIV-infected patients compared with the controls. For example, the control subjects appeared to have higher levels of bacteria of the *Bacteroidetes* (77.58% relative abundance) than the chronic HIV-infected patients (14.78%). Within this phylum, the change in relative abundance was most prominent for the genera *Parabacteroides*, *Bacteroides* and others belonging to the class *Bacteroidia*, including taxa in *Porphyromonadacea*e, *Bacteroidaceae* and others in the order *Bacteroidales.*

We are not the first to consider the relationship between HIV infection and the fecal bacterial microbiome. A recent report by Dinh *et al.*,^21^ which compared the fecal microbial community composition of patients suffering from HIV with that of healthy controls, presented results that are somewhat different from ours. In that study, the relative abundances of *Proteobacteria, Gammaproteobacteria, Enterobacteriales, Enterobacteriaceae, Erysipelotrichi, Erysipelotrichales, Erysipelotrichaceae* and *Barnesiella* were significantly higher in HIV patients, whereas those of *Rikenellaceae* and *Alistipes* were lower. The lack of corroboration between this prior study and the current study is surprising as similar methodologies with regard to subject enrollment, sample DNA extraction, 16S rRNA gene amplification and 454 pyrosequencing were used in both studies. However, these two studies differed primarily in terms of the patient populations recruited for evaluation. The present study focused on Chinese subjects, whereas Dinh *et al.*^21^ enrolled American subjects. Lozupone *et al.*^22^ also sought to determine the association between HIV infection and characteristic fecal bacterial microbiome changes and found that the microbiome of HIV-infected individuals in the United States was similar to that of healthy individuals residing in the agrarian cultures of Malawi and Venezuela. The researchers related this phenomenon to the diets of these individuals, which are carbohydrate-rich and poor in protein and fat. Overall, these findings indicate that the bacterial composition and balance in the fecal bacterial microbiome is dependent not only on the health status of the individual but also on their environment and diet, which may partially explain the difference in the observed microbiome changes between American and Chinese subjects with HIV infection.

Additional factors affecting the fecal microbiome, including alcohol use, lean body mass, nutritional factors, and the intake of carbohydrate-rich, protein-poor and fat-poor diets, have also been investigated.^23, 24, 25, 26, 27^ Volpe *et al.*^23^ focused on cocaine-induced differences in the relative abundance of major phyla in the fecal bacterial microbiome of HIV-infected patients, as well as on markers of inflammation and microbial translocation. In that study, HIV-infected individuals similar to the Chinese subjects in our study had a higher relative abundance of *Proteobacteria,* whereas cocaine users had a higher relative abundance of *Bacteroidetes.* Furthermore, HIV-infected cocaine users had the highest interferon levels indicating that drug use may further alter the microbiome and exacerbate inflammation.

We present the first epidemiologic study comparing the fecal bacterial microbiome of chronic HIV-infected patients with that of non-HIV infected controls in China. Chinese HIV-infected patients are similar to the subjects in other parts of the world in that HIV infection reduces microbiome diversity and enriches *Proteobacteria*. However, the affected lower ranked taxa in *Proteobacteria* were different between the two populations. Because most of our control subjects had diarrhea, the difference could be caused by diarrhea. Dietary factors may also play a role. Because the enrichment of *Proteobacteria* is also a common finding in inflammatory states,^28^ similar changes in HIV infection may play a functional role in regulating innate immunity, inflammation and disease progression. The unique microbiome change in Chinese HIV-infected patients should be further investigated to better understand its impact on disease progression and response to ART. An increased understanding of the correlations between the proinflammatory bacterial community composition in the human fecal bacterial microbiome and chronic HIV infection-related health complications will contribute to improved and holistic management of this disease.

Our study was limited by a small sample size. In particular, our study was subject to type-II error (that is, failing to detect an effect that is present in a small scale study). The power of this study was further weakened by a heterogeneous study population that varied in gender, antibiotic use and HIV progression. Thus, a large-scale study is needed to confirm our findings.

## Figures and Tables

**Table 1:** Demographic and clinical characteristics of the study subjects who provided fecal specimens

	**Sample ID**	**Gender**	**Age**	**ART**^a^	**CD4 (cell/mm**^**3**^)	**Viral load (copy/mL)**^b^	**Antibiotic use**^c^	**Complications**^d^
**Chronic HIV-infected patients** (*n*=13)	HIV3	M	24	HAART	788	66100	Yes	PLA
	HIV4	F	22	HAART	222	ND	Yes	PI/Syp
	HIV5	M	30	No	11	ND	No	PI/ NTM
	HIV6	F	48	No	756	ND	Yes	No
	HIV7	M	54	HAART	30	ND	No	PI/NTM/CMVI
	HIV8	M	72	HAART	75	ND	No	Lymp
	HIV9	M	47	HAART	26	ND	No	PI
	HIV10	M	58	HAART	158	46400	Yes	PLA/CDA
	HIV11	M	24	HAART	38	71	No	PI
	HIV12	F	27	HAART	36	716	Yes	PI/FI
	HIV13	F	34	HAART	414	731	Yes	No
	HIV14	M	32	HAART	484	1720	No	No
	HIV15	M	38	HAART	328	2500	No	No
	NT10	M	46	No	ND^e^	ND	No	No
								
**Non-HIV controls** (*n*=7)	NT5	M	46	No	ND	ND	No	No
	NT7	M	46	No	ND	ND	No	No
	NT9	M	46	No	ND	ND	No	No
	DB3	F	29	No	ND	ND	No	Diar
	DP3	F	66	No	ND	ND	No	Diar
	DP4	M	71	No	ND	ND	No	Diar

a antiretroviral therapy, ART; number of sequence reads, Read No.

b Viral load: copy/mL.

c Antibiotic use four weeks prior to fecal sampling.

d pulmonary infection, PI; syphilis, Syp; pyogenic liver abscess, PLA; non-tuberculosis mycobacteria, NTM; CMV infection, CMVI; lymphoma, Lymp; clinical diagnosis of amoeba, CDA; fungal infection, FI; diarrhea, Diar.

e not done, ND.

**Table 2:** *Genus*-level differences between chronic HIV-infected patients and non-HIV infected controls

**OTU**	P**-value**	**Bonferroni-corrected**	**FDR-corrected**	**Control mean**	**HIV+mean**
*Parabacteroides*	3.00E-07	2.76E-05	2.76E-05	1.05E-01	2.51E-03
*Bacteroides*	6.89E-07	6.34E-05	3.17E-05	5.23E-01	2.16E-02
*Lachnospiraceae-*unclassified	8.42E-07	7.74E-05	2.58E-05	6.85E-03	4.43E-04
*Sutterella*	8.52E-07	7.84E-05	1.96E-05	1.52E-02	1.61E-04
*Phascolarctobacterium*	1.30E-06	1.20E-04	2.40E-05	7.96E-02	9.81E-05
*Faecalibacterium*	3.70E-06	3.40E-04	5.67E-05	6.54E-02	1.96E-03
*Clostridia-*unclassified	4.57E-06	4.20E-04	6.00E-05	1.30E-03	2.00E-05
*Bacteroidales-*unclassified	2.77E-04	2.55E-02	3.18E-03	1.07E-02	5.21E-04
*Roseburia*	3.82E-04	3.51E-02	3.90E-03	1.17E-02	1.36E-03
*Bacteroidaceae-*unclassified	6.15E-04	5.66E-02	5.66E-03	2.37E-02	6.84E-04
*Lachnospira*	8.08E-04	7.43E-02	6.76E-03	3.57E-02	5.73E-04
*Bacteroidetes-*unclassified	2.01E-03	1.85E-01	1.54E-02	9.20E-05	9.96E-06
*Clostridiales-*unclassified	4.57E-03	4.21E-01	3.24E-02	1.52E-03	2.88E-04
*Streptococcus*	2.12 E-01	19.52	4.76 E-01	6.12 E-04	3.58 E-02
*Pseudomonas*	2.32 E-01	21.31	4.63 E-01	4.88E-06	8.67 E-02
*Sphingobacterium*	1.00 E-01	9.26	4.20 E-01	4.48E-05	9.69 E-02
*Bilophila*	7.13E-03	6.56E-01	4.69E-02	2.78E-03	0

Abbreviation: OTU, Operational taxonomic unit.

The taxonomic classification (genus level) and taxa abundance matrix were analyzed with the QIIME pipeline. Differences between the two experimental groups were analyzed by ANOVA followed by *post-hoc* tests with Bonferroni correction and FDR correction (*q*-values). *P*<0.05 and *q*<0.1 were considered significant.
