# Supplementary material for: Fecal bacterial microbiome diversity in chronic HIV-infected patients in China
Source: Emerg Microbes Infect. 2016 Apr 6;5(4):e31–. doi: 10.1038/emi.2016.25 (PMC4855070; doi:10.1038/emi.2016.25)
Supplement: Supplementary Table S1 [file emi201625x1.pdf]

Supplementary Table S1 Phylum-level taxonomic differences between chronic HIV-infected patients and non-HIV infection controls

OTU	*P*-value	Bonferroni-corrected	FDR-corrected	Control mean	HIV+ mean
k__Bacteria;p__Bacteroidetes	1.52E-07	9.13E-07	9.13E-07	0.775813989	0.147832012
k__Bacteria;Other	0.008380059	0.050280352	0.025140176	0.003836364	0.001659252
k__Bacteria;p__Proteobacteria	0.03138889	0.188333338	0.062777779	0.037923831	0.371996573
k__Bacteria;p__Firmicutes	0.104793493	0.628760958	0.15719024	0.179438158	0.471961368
k__Bacteria;p__Fusobacteria	0.147196157	0.88317694	0.176635388	0.001973902	0.000479452
k__Bacteria;p__Actinobacteria	0.165713775	0.994282648	0.165713775	0.000966363	0.00589976
