# Supplementary material for: Fecal bacterial microbiome diversity in chronic HIV-infected patients in China
Source: Emerg Microbes Infect. 2016 Apr 6;5(4):e31–. doi: 10.1038/emi.2016.25 (PMC4855070; doi:10.1038/emi.2016.25)
Supplement: Supplementary Table S2 [file emi201625x2.pdf]

Supplementary Table S2 Genus-level distribution of higher taxa in chronic HIV-infected patients and non-HIV infection controls

OTU	prob	Bonferroni_corrected	FDR_corrected	N_mean	hiv_mean
k__Bacteria;p__Bacteroidetes;c__Bacteroidia;o__Bacteroidales;f__Porphyromonadaceae;g__Parabacteroides	3.00E-07	2.76E-05	2.76E-05	0.10477761	0.002511731
k__Bacteria;p__Bacteroidetes;c__Bacteroidia;o__Bacteroidales;f__Bacteroidaceae;g__Bacteroides	6.89E-07	6.34E-05	3.17E-05	0.523052356	0.021675361
k__Bacteria;p__Firmicutes;c__Clostridia;o__Clostridiales;f__Lachnospiraceae;g__	8.42E-07	7.74E-05	2.58E-05	0.006853428	0.000442636
k__Bacteria;p__Proteobacteria;c__Betaproteobacteria;o__Burkholderiales;f__Alcaligenaceae;g__Sutterella	8.52E-07	7.84E-05	1.96E-05	0.015221217	0.000161147
k__Bacteria;p__Firmicutes;c__Clostridia;o__Clostridiales;f__Veillonellaceae;g__Phascolarctobacterium	1.30E-06	0.000120011	2.40E-05	0.007959459	9.81E-05
k__Bacteria;p__Firmicutes;c__Clostridia;o__Clostridiales;f__Ruminococcaceae;g__Faecalibacterium	3.70E-06	0.000340433	5.67E-05	0.065438871	0.001961447
k__Bacteria;p__Firmicutes;c__Clostridia;o__;f__;g__	4.57E-06	0.000420327	6.00E-05	0.001301802	2.00E-05
k__Bacteria;p__Bacteroidetes;c__Bacteroidia;o__Bacteroidales;Other;Other	0.000276806	0.025466125	0.003183266	0.010678872	0.000520961
k__Bacteria;p__Firmicutes;c__Clostridia;o__Clostridiales;f__Lachnospiraceae;g__Roseburia	0.000381639	0.035110814	0.003901202	0.011709869	0.00135755
k__Bacteria;p__Bacteroidetes;c__Bacteroidia;o__Bacteroidales;f__Bacteroidaceae;Other	0.000614674	0.056550026	0.005655003	0.023739064	0.000683761
k__Bacteria;p__Firmicutes;c__Clostridia;o__Clostridiales;f__Lachnospiraceae;g__Lachnospira	0.000807989	0.074334969	0.006757724	0.035709688	0.000573428
k__Bacteria;p__Bacteroidetes;Other;Other;Other;Other	0.002011821	0.185087501	0.015423958	9.20E-05	9.96E-06
k__Bacteria;p__Firmicutes;c__Clostridia;o__Clostridiales;Other;Other	0.004571561	0.420583568	0.032352582	0.001519606	0.000287655
k__Bacteria;p__Proteobacteria;c__Deltaproteobacteria;o__Desulfovibrionales;f__Desulfovibrionaceae;g__Bilophila	0.007132287	0.656170422	0.046869316	0.002782059	0
